# Attachment of Cancer Urothelial Cells to the Bladder Epithelium Occurs on Uroplakin-Negative Cells and Is Mediated by Desmosomal and Not by Classical Cadherins

**DOI:** 10.3390/ijms22115565

**Published:** 2021-05-25

**Authors:** Urška Dragin Jerman, Tanja Višnjar, Iva Hafner Bratkovič, Nataša Resnik, Mojca Pavlin, Peter Veranič, Mateja Erdani Kreft

**Affiliations:** 1 Institute of Cell Biology, Faculty of Medicine, University of Ljubljana, 1000 Ljubljana, Slovenia; urska.dragin@mf.uni-lj.si (U.D.J.); tanja.visnjar@gmail.com (T.V.); natasa.resnik@mf.uni-lj.si (N.R.); 2 National Institute of Chemistry, 1000 Ljubljana, Slovenia; iva.hafner@KI.si; 3 En-FIST Centre of Excellence, 1000 Ljubljana, Slovenia; 4 Group for Nano and Biotechnological Applications, Faculty of Electrical Engineering, University of Ljubljana, 1000 Ljubljana, Slovenia; Mojca.Pavlin@fe.uni-lj.si; 5 Institute of Biophysics, Faculty of Medicine, University of Ljubljana, 1000 Ljubljana, Slovenia

**Keywords:** bladder cancer, urothelial *in vitro* models, N-cadherin, desmosomes, electron microscopy

## Abstract

Urinary bladder cancer is often multifocal; however, the intraluminal dissemination of the urothelial cancer cells is poorly understood. The involvement of N-cadherin in the adhesion of the cancer urothelial cells to the urothelium had not previously been studied. Therefore, we herein explore the possibility of the intraluminal dissemination of the urothelial cancer cells by evaluating the role of classical cadherins in the adhesion of urothelial cancer cells to the urothelium. We used E-cadherin negative T24 cells and established a T24 Ncad^low^ cell line with an additionally decreased expression of N-cadherin in the plasma membrane and a decreased secretion of proform of metalloproteinase 2. The labelled T24 and T24 Ncad^low^ cells were seeded onto urothelial in vitro models. After 24 h in co-culture, unattached cancer cells were rinsed and urothelia with attached cancer urothelial cells were processed for fluorescence and electron microscopy. Both the T24 and T24 Ncad^low^ cells attached to the urothelium, yet only to the uroplakin-negative urothelial cells. The ultrastructural analysis showed that T24 and T24 Ncad^low^ cells adhere to poorly differentiated urothelial cells by desmosomes. To achieve this, they first disrupt tight junctions of superficial urothelial cells. This study indicates that the lack of E-cadherin expression and decreased expression of N-cadherin in the plasma membrane of T24 cells does not interfere with their adhesion to the urothelium; therefore, our results suggest that intraluminal dissemination of cancer urothelial cells along the urothelium occurs on uroplakin-negative cells and is desmosome-mediated.

## 1. Introduction

Bladder cancer ranks as the ninth most frequently diagnosed cancer worldwide, with the highest incidence rates observed in men in Southern and Western Europe and North America [1]. The bladder tumors are highly recurrent and are often multifocal [2]. The mechanisms of bladder tumor recurrence or multifocality are not yet clear and are being interpreted by different theories. The theory of field cancerization proposes that multiple and recurrent tumors arise due to independent genetic events in individual urothelial cells caused by carcinogens, meaning they are not clonally related [3]. Alternatively, the clonal theory suggests that multifocal/recurrent tumors result from dissemination of cancer urothelial cells from the primary tumor either by migration within the urothelium [4] or by release from the primary tumor into bladder lumen and implantation in the urothelium at a distant site [5]. The majority of molecular and genetic studies have demonstrated the clonal origin of bladder tumors [5,6,7,8,9,10]. Nevertheless, cells in the clonal relationship may not necessarily share the mutation profile completely; therefore, determination of the exact origin of the tumors could be challenging [11]. However, the proposed theories are not mutually exclusive, since both the clonally and non-clonally related tumors may coexist in the same patient [12].

This study explores the possibility of the intraluminal dissemination of urothelial cancer cells, with the focus on cell junctions through which cancer urothelial cells attach to the urothelium. Previous studies that addressed related issues compared the adhesion of urothelial cancer cells to the normal urothelium, depending on their expression of classical cadherins [13,14,15]. In epithelial cells, cadherins mediate cell–cell interactions within highly ordered junctional complexes: the adherens junctions and desmosomes [16]. The studies report that adhesion of E-cadherin-expressing cancer urothelial cells to the normal urothelium could be E-cadherin-dependent since such cancer urothelial cells adhered to the normal urothelium [13,14,15]. However, during progressive cancer transformation, epithelial cells often switch the expression from E-cadherin to N-cadherin [17]. The cadherin switch causes changes in epithelial cell phenotype, meaning that epithelial cells become more fibroblast-like and thus more motile [18]. Accordingly, various studies demonstrate the crucial involvement of N-cadherin in cancer cell invasion, collective migration, and metastasis (reviewed in [19]). It has been shown that N-cadherin can promote adhesion of melanoma and breast cancer cells to the endothelium [20,21]. Nevertheless, to the best of our knowledge, the involvement of N-cadherin in the adhesion of the cancer urothelial cells to the urothelium has not been studied. Additionally, none of the performed studies directly showed or identified cell junctions involved in the adhesion of cancer urothelial cells to the urothelium. 

Cadherins are homotypic binding molecules. Yet, an increasing number of studies show that cadherins can also mediate cell–cell adhesion through heterotypic interactions [22,23,24,25]. This suggests that N-cadherin-expressing cancer urothelial cells may associate with E-cadherin-expressing urothelial cells via E-N cadherin junctions. In this study, we investigated the possible role of N-cadherin in the adhesion of cancer urothelial cells to the urothelia at different stages of differentiation. We have shown that this adhesion is not crucially mediated by N-cadherin but instead involves the desmosomes. 

## 2. Results

### 2.1. Urothelial In Vitro Models: Poorly Differentiated Urothelia from RT4 Cells and Partially Differentiated Urothelia Established from Normal Urothelial (NPU) Cells

**Figure 1:**
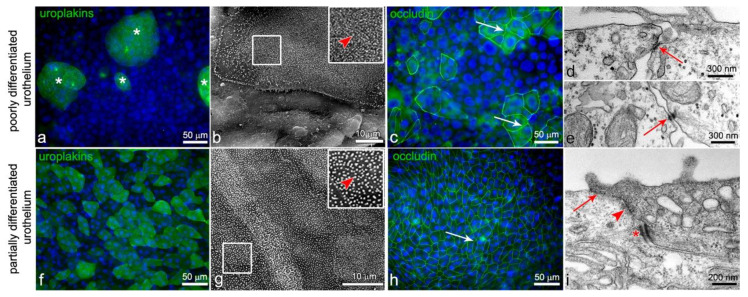
(**a**–**e**) After 1 week in culture, RT4 cells form poorly differentiated urothelium. Only a few cells in the superficial layer are partially differentiated and express differentiation-related uroplakins in the apical plasma membrane (asterisks on (**a**)). The apical surface of these cells is shaped in microvilli (arrowhead on (**b**)) and they express occludin in the lateral plasma membranes (arrows on (**c**)). The majority of urothelial cells in the superficial layer are poorly differentiated and do not express uroplakins (**a**) or interconnect with tight junctions (**c**). Nevertheless, they interconnect with adherens junctions (arrow on (**d**)) or/and desmosomes (arrow on (**e**)). After 1 week in culture, NPU cells form partially differentiated urothelium with partially differentiated superficial urothelial cells (**f**–**i**). The vast majority of them express uroplakins (**f**) and have an apical plasma membrane still shaped into microvilli (arrowhead on (**g**)). The superficial cells interconnect by tight junctions (arrows on (**h**,**i**)), adherens junctions (arrowhead on (**i**)), and desmosomes (asterisk on (**i**)). Nuclei are stained blue with DAPI. The areas in the smaller frames on (**b**,**g**) are enlarged by 100%. No signal was detected on immunofluorescence negative controls. Immunofluorescence (**a**,**c**,**f**,**h**); scanning electron microscopy (**b**,**g**); and transmission electron microscopy (**d**,**e**,**i**).

As an *in vitro* model of poorly differentiated urothelium human non-invasive cancer urothelial cells, RT4, isolated from transitional cell papilloma were used. The RT4 cells formed two-to-three-layered urothelium with two types of urothelial cells in the superficial layer that were at different stages of differentiation. The majority of urothelial cells in the superficial layer were poorly differentiated, as they did not express uroplakins and did not interconnect with occludin-positive tight junctions (Figure 1a–e). Among them, were partially differentiated urothelial cells with an apical plasma membrane shaped into microvilli, uroplakins on the apical surface, and established tight junctions (Figure 1a–c). As an *in vitro* model of the partially differentiated urothelium, we used secondary cell cultures of normal porcine urothelial (NPU) cells. After 5 to 7 days in culture, the NPU cells established two-to-three-layered urothelium with partially differentiated superficial urothelial cells. Most of these cells expressed uroplakins in the apical plasma membrane although, in varying degrees, with individual superficial cells being uroplakin negative. The apical plasma membrane of superficial urothelial cells was mainly shaped into microvilli (Figure 1g) and the cells were interconnected by occludin-positive tight junctions (Figure 1h,i).

### 2.2. T24 Ncad^low^ Cells Are Characterized by Lower Expression of N-Cadherin in the Plasma Membrane Compared with T24 Cells

**Figure 2:**
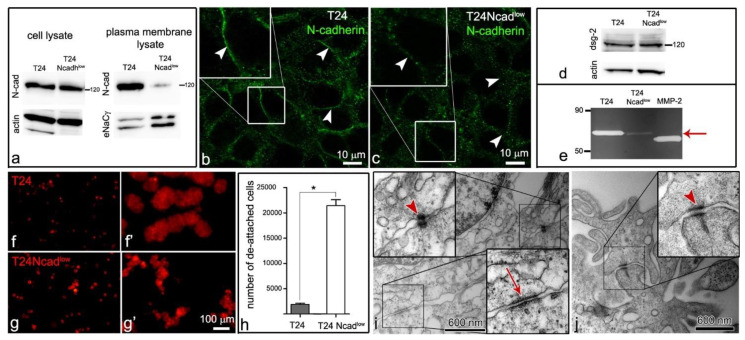
(**a**) The expression of N-cadherin in the plasma membrane of T24 Ncad^low^ cells is lower compared with its expression in the T24 plasma membrane. eNaCγ is presented as loading control for the plasma membrane proteins and actin is presented as a protein loading control. Note that although a load of plasma membrane proteins was higher in the case of T24 Ncad^low^ cells, the expression of N-cadherin in their plasma membrane was still lower compared with the T24 cells. (**b**,**c**) The distribution of N-cadherin in T24 Ncad^low^ cells is punctuated (arrowheads on (**c)**) and not arranged in a thin line as it was between the neighboring T24 cells (arrowheads on (**b**)). (**d**) Both the T24 and T24 Ncad^low^ cells express desmoglein-2, the desmosomal cadherin. (**e**) The gel gelatin zymogram shows that T24 cells secrete more proform of metalloproteinase 2 (pro-MMP-2; 72 kDa) than T24 Ncad^low^ cells (arrow). The proteolytic activity of the pro-MMP 2 and MMP2 is visualized as white bands on a dark background. MMP-2 in the third line is a recombinant MMP-2 standard protein (active form, 68 kDa). (**f**–**g’**) After 24 h in suspension, the T24 cells (**f’**) form large and compact cell aggregates. The aggregates of T24 Ncad^low^ cells (**g’**) are smaller and less compact. Images (**f**,**g**) show cells at the establishment of cell suspension, while **f’**,**g’** show the same cell culture in suspension after 24 h. The T24 and T24 Ncad^low^ are labeled red with a lipophilic dye. (**h**) The hanging drop assay confirms that T24 cells are more tightly interconnected compared with T24 Ncad^low^ cells. The graph shows an average number of cells that were detached from the cell cluster after the mechanical stimulus ± SEM, * *p* < 0.001. (**i,j**) T24 and T24 Ncad^low^ cells within the aggregate interconnect with desmosomes (arrowheads on (**i**,**j**)). On the ultrastructural level, we found adherens junctions only among the T24 cells (arrow on (**i**)). The areas in the smaller frames are enlarged by 100%. No signal was detected on immunofluorescence negative controls. Immunofluorescence (**b**,**c**,**f**–**g’**) and transmission electron microscopy (**i**,**j**).

As an *in vitro* model of cancer urothelial cells, we used human invasive cancer urothelial cells, T24, isolated from transitional cell carcinoma, and T24 Ncad^low^ cells, which were established *de novo* from the T24 cell line (described in Section 4.2). Biotinylation of the T24 and T24 Ncad^low^ cell plasma membranes revealed that T24 Ncad^low^ cells expressed significantly less N-cadherin in the plasma membrane compared with the T24 cells (Figure 2a–c). The quantification of N-cadherin band intensity demonstrated a 93.5% decrease in N-cadherin expression in the plasma membrane of T24 Ncad^low^ cells (0.172 ± 0.03 a.u.) compared with the T24 cells (2.651 ± 0.67 a.u., *p* < 0.01). Accordingly, the distribution of N-cadherin at the lateral plasma membrane of T24 Ncad^low^ cells was punctuated and not arranged in a thin continuous line as it was at the interjunctions of the T24 cells (Figure 2b,c). The T24 and T24 Ncad^low^ did not express E-cadherin, while the RT4 cells of poorly differentiated urothelium and NPU cells of partially differentiated urothelium were E-cadherin positive (Figure 2d). The T24 and T24 Ncad^low^ both expressed desmoglein-2, the desmosomal cadherin (Figure 2d), and were shown to interconnect with desmosomes (Figure 2i,j). Moreover, the T24 Ncad^low^ secreted less pro-MMP2 gelatinase in the culture medium than T24 cells (Figure 2e). 

When seeded onto the poorly or partially differentiated urothelium, the T24 Ncad^low^ cells formed small and less compact aggregates than T24 cells (Figure 2f–g’). Diminished intercellular adhesion of cancer urothelial cells T24 Ncad^low^ was additionally confirmed by hanging drop assay, where significantly more T24 Ncad^low^ cells (35.7 ± 2%) than T24 cells (3.2 ± 0.37%) were released from the cell aggregates after the mechanical stimulus (Figure 2h).

### 2.3. The Lower Expression of N-Cadherin in the Plasma Membrane of the T24 Ncad^low^ Cells Does Not Affect Their Attachment to the Poorly or Partially Differentiated Urothelium

**Figure 3:**
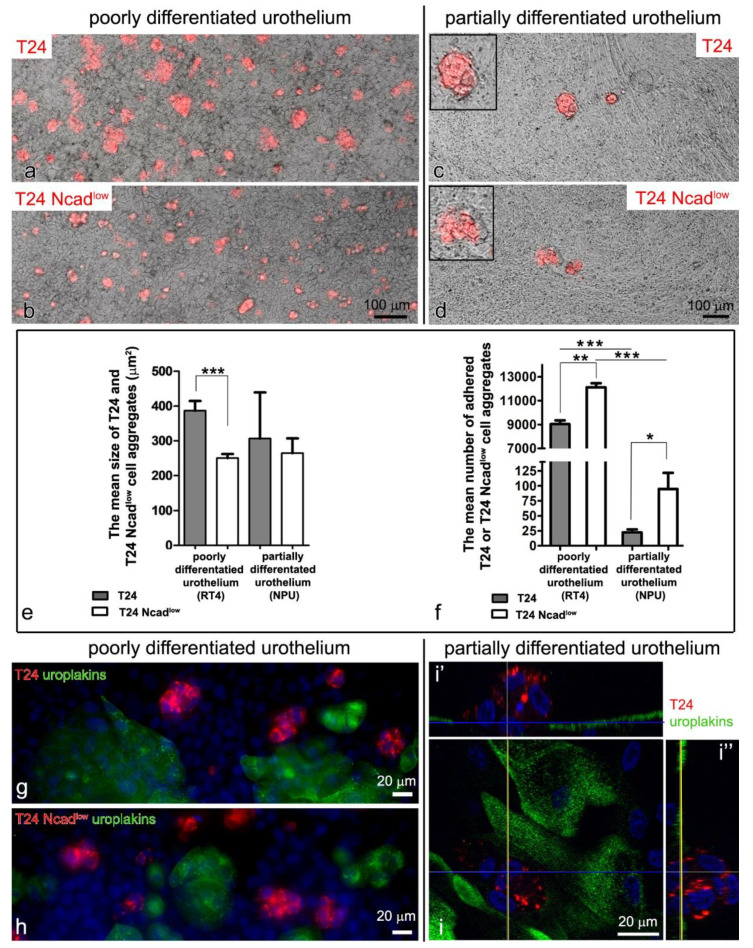
Attachment of T24 and T24 Ncad^low^ cells to the poorly and partially differentiated urothelium, 24 h after seeding. (**a**–**d**) The T24 and T24 Ncad^low^ cells are labeled red with a lipophilic dye. The T24 and T24 Ncad^low^ cells attach to the poorly (**a**,**b**) or partially (**c**,**d**) differentiated urothelia as cell aggregates. The aggregates of T24 cells are large and compact (**a**,**c**), while T24 Ncad^low^ cells form smaller aggregates (**b**,**d**). The areas in the frames on (**c**,**d**) are enlarged by 50%. (**e**) The graph shows the mean size of T24 and T24 Ncad^low^ cell aggregates adhered to the poorly or partially differentiated urothelium ± SEM, (*** *p* < 0.001). The T24 Ncad^low^ cell aggregates adhered to poorly differentiated urothelium were significantly smaller than T24 cell aggregates. Note that the trend is the same in the partially differentiated urothelium, although the difference is not statistically significant. This is due to the overall small number of cancer cell aggregates that adhered to partially differentiated urothelium. (**f**) The graph demonstrates the mean number of T24 and T24 Ncad^low^ cell aggregates attached to the poorly or partially differentiated urothelium per cm^2^ area ± SEM, (* *p* < 0.05, ** *p* < 0.01, *** *p* < 0.001). After 24 h, significantly more aggregates are adhered to poorly than partially differentiated urothelium. Although both cell lines were seeded with the same seeding density, a significantly higher number of smaller T24 Ncad^low^ cell aggregates was found on the surface of the urothelium, possibly due to their less intense aggregation. (**g**–**i**) The T24 and T24 Ncad^low^ cells attach only to the uroplakin negative urothelial cells. The blue line on (**i**) indicates the intersection of cells shown in (**i’**), and yellow line on (**i**) indicates the intersection of cells shown in (**i’’**). Nuclei are stained blue with DAPI. No signal was detected on immunofluorescence negative controls. Phase-contrast microscopy (**a**–**d**) and immunofluorescence (**a**–**d**,**g**–**i’’**).

The T24 and T24 Ncad^low^ cells were left to adhere to the *in vitro* models of poorly or partially differentiated urothelium for 24 h. In the vast majority, both cell types had already aggregated in culture media and adhered to the poorly or partially differentiated urothelium as cell aggregates (Figure 3a–d). The T24 cell aggregates adhered to the poorly differentiated urothelia and on average covered 386.7 ± 28 µm^2^ surface area and were significantly larger than the T24 Ncad^low^ cell aggregates (250.7 ± 11.7 µm^2^). The trend was the same in cancer cell aggregates adhered to the partially differentiated urothelia with the T24 cell aggregates of 307.6 ± 132.4 µm^2^ size and T24 Ncad^low^ cell aggregates of 264.6 ± 95.3 µm^2^ size (Figure 3e).

Overall, 9038 ± 313.3 T24 aggregates and 12,126 ± 335.1 T24 Ncad^low^ aggregates attached to the cm^2^ of poorly differentiated urothelia in 24 h (*p* < 0.01; Figure 3f). In the case of the partially differentiated urothelia, the number of attached cancer cell aggregates in 24 h was significantly lower: 22.5 ± 4.9 aggregates of T24 cells and 94.6 ± 26.9 aggregates of T24 Ncad^low^ cells per cm^2^ (*p* < 0.05; Figure 3f). 

RT4 and NPU cells were cultured in different culture media (A-DMEM + F12 and UroM (−Ca^2+^ + S_FBS_), respectively) to maintain the poorly and partially differentiated urothelial models. The T24 and T24 Ncad^low^ cells were seeded onto different urothelial models in their assigned culture medium. To demonstrate that the culture medium itself did not affect the adhesion of cancer urothelial cells to the urothelium, we also seeded T24 and T24 Ncad^low^ cells to the poorly differentiated urothelium in UroM (−Ca^2+^ + S_FBS_) culture media. The mean number of the adhered urothelial cancer cell to the poorly differentiated urothelium in the UroM (−Ca^2+^ + S_FBS_) culture medium was 9389 ± 254.6 for T24 cell aggregates and 10,429 ± 269.6 for T24 Ncad^low^ cell aggregates per cm^2^. This means that significantly more T24 and T24 Ncad^low^ cell aggregates adhered to the poorly differentiated urothelium than to the partially differentiated urothelium in the UroM (−Ca^2+^ + S_FBS_) culture medium (Figure 3e), further indicating that the effect of the culture medium on the adhesion of cancer urothelial cells to the urothelium was negligible. 

**Figure 4:**
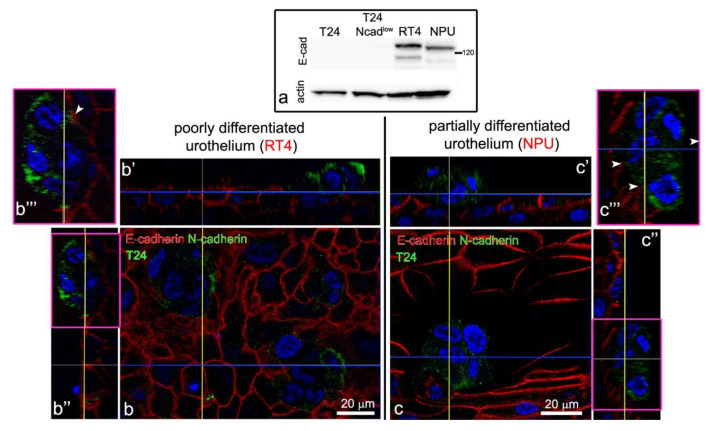
Expression of E- and N-cadherin in poorly and partially differentiated urothelium with attached T24 cell aggregates, 24 h after their seeding. (**a**) Urothelial cells of poorly (RT4 cells) and partially (NPU cells) differentiated urothelium express E-cadherin, while T24 and T24 Ncad^low^ cells are E-cadherin negative. (**b**,**c**) T24 cells express N-cadherin (green) in the plasma membrane, however, there is no co-localization of E- (red) and N-cadherin (green) signals at contact sites of the T24 cells and the urothelial cells of poorly (**b**) or partially (**c**) differentiated urothelium (arrowheads on (**b’’’**) (enlarged **b’’**) and (**c’’’**) (enlarged (**c’’**)). The blue and yellow lines on (**b**,**c**) indicate the intersections of cells shown in (**b’,c’** and **b’’,c’’**), respectively. The area in the smaller frame is enlarged by 100%. Nuclei are stained blue with DAPI. No signal was detected on immunofluorescence negative controls. Immunofluorescence (**b**–**c’’’**).

**Figure 5:**
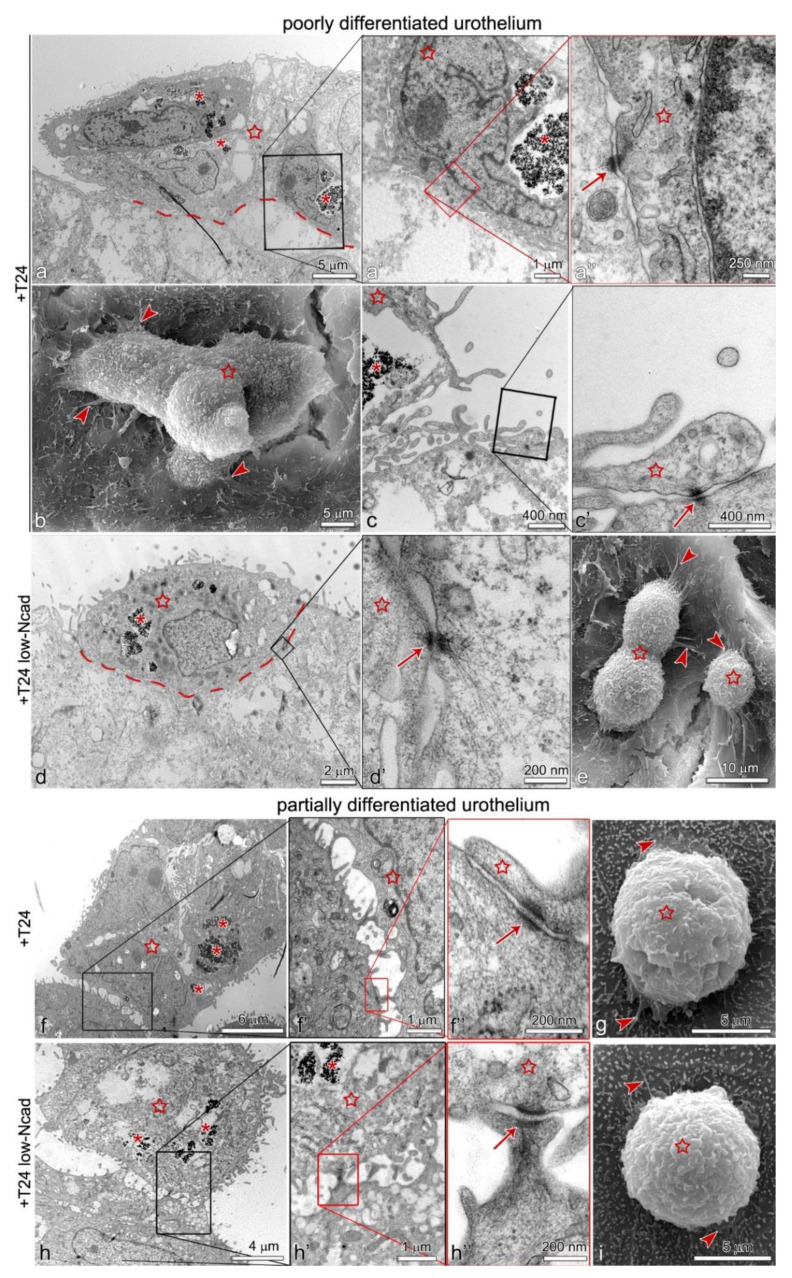
Ultrastructure of T24 and T24 Ncad^low^ cell aggregates attached to the poorly (**a**–**e**) and partially (**f**–**i**) differentiated urothelium. T24 and T24 Ncad^low^ cells are labeled with PAA nanoparticles (asterisks on (**a,a’,d,f,h,h’**)). (**a**–**c’**,**f**–**g**) T24 cells (star) attach to the poorly and partially differentiated urothelium in aggregates in (**a**). The individual T24 cells from the aggregate adhere to the urothelial cells by desmosomes, which are recognized by dense plaques and tethering to intermediate filaments (enlarged on (**a’**) and (**a’’**,**f’’**) (arrow)). At the edge of the aggregate, the T24 cells form lamellipodia and filopodia (arrowheads on (**b**,**g**)). Note that T24 cells adhere to the urothelial cells by desmosomes also on the tips of the filopodia ((**c**), arrow on (**c’**)). (**d**–**e,h**–**i**) The cells at the base of T24 Ncad^low^ cell aggregates (marked with a star) also attach to the urothelial cells of the poorly and partially differentiated urothelium with desmosomes (arrow on (**d’**,**h’’**)). At adhesion, the T24 Ncad^low^ cells, similarly to T24 cells, form lamellipodia and filopodia (arrowheads on (**e**,**i**)). Transmission electron microscopy (**a**–**a’’**,**c**–**d’**,**f**–**f’’**,**h**–**h’’**) and scanning electron microscopy (**b**,**e**,**g**,**i**).

**Figure 6:**
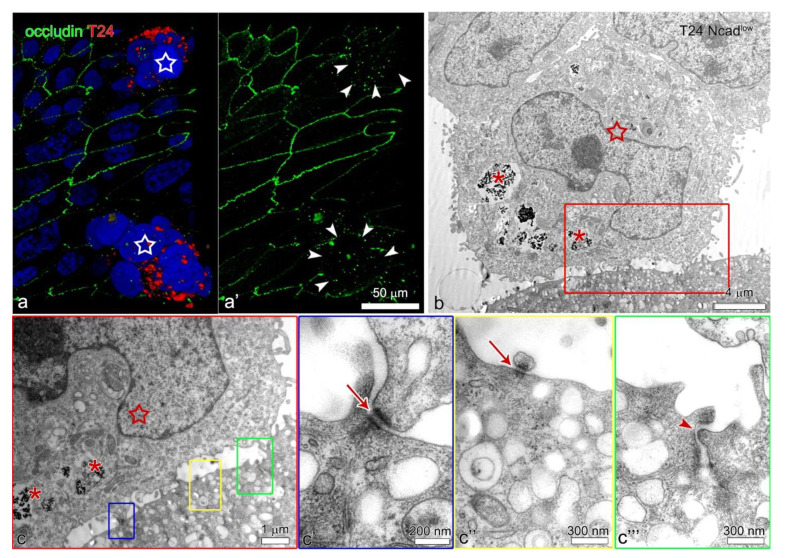
The urothelial cells in the superficial layer of partially differentiated urothelium with attached T24 and T24 Ncad^low^ cell aggregates have disrupted tight junctions. (**a,a’**) 3D model of T24 cell aggregates adhered to the partially differentiated urothelium (image **a**: red and green channel, image (**a’**): green channel). T24 cells (star) are labeled red with a lipophilic dye. Note the disrupted tight junctions of urothelial cells underneath the T24 cell aggregates (area marked with arrowheads on (**a’**)). (**b**–**c’’’**) T24 Ncad^low^ cells are labeled with PAA nanoparticles (asterisks on (**b**,**c**)) and marked with a star. T24 Ncad^low^ cells adhered to the partially differentiated urothelium with desmosomes (enlarged in (**c’**,**c’’**), arrows). Note that the tight junctions of urothelial cells underneath T24 Ncad^low^ cells are loosened (arrowhead on (**c’’’**)). No signal was detected on immunofluorescence negative controls. Immunofluorescence: (**a**,**a’**); Transmission electron microscopy: (**b**–**c’’’**).

Both, the T24 and T24 Ncad^low^ cells only attached to the uroplakin negative urothelial cells (Figure 3f–h’’). By co-immunolabelling of E- and N-cadherin we did not identify heterotypical E- and N-cadherin junctions between the T24 cells and urothelial cells in the superficial layer of the poorly or partially differentiated urothelium (Figure 4b–c’’’). Further, we did not observe adherens junctions between the T24 or T24 Ncad^low^ cells and urothelial cells on an ultrastructural level. Conversely, the ultrastructural analysis of the attachment sites showed that T24 and T24 Ncad^low^ cells at the base of the cell aggregate attached to the urothelial cells in the superficial layer of poorly or partially differentiated urothelium by desmosomes (Figure 5). The urothelial cells underneath the adhered T24 and T24 Ncad^low^ cell aggregates had disrupted tight junctions (Figure 6).

## 3. Discussion

Multifocality and high recurrence of bladder tumors are two main frustrations and concerns of clinical urologists. To design effective prevention and treatment strategies, it is essential to understand the cell biological processes leading to these pathologies. Several theories try to explain bladder cancer progression or recurrence [3,11]. One theory suggests that multiple and recurrent bladder tumors could result from the intraluminal dissemination of viable cancer urothelial cells from the primary tumor. Nevertheless, the molecules and mechanisms involved in cancer urothelial cell attachment to the urothelium have not yet been identified. In this study, we investigated the possible involvement of N-cadherin in the adhesion of cancer urothelial cells T24 to the urothelium. 

It is estimated that around 75% of patients with bladder cancer diagnosis present non-muscle-invasive superficial tumors (Ta, T1, or tumors in situ) [26], which are commonly multifocal [27]. The multifocal tumors may arise synchronously [6] or more often after resection of the primary tumor [28]. The Ta papillary tumors are usually low-grade tumors, whereas tumors in situ and most of the T1 papillary tumors are high-grade tumors, and in 30% to 60% eventually progress in stage [29,30]. N-cadherin is expressed in up to 40% of non-muscle-invasive Ta and T1 bladder tumors [31,32,33], and its expression in these tumors is associated with a higher probability of tumor recurrence [32]. To illustrate the situation of the high-grade T1 tumor with N-cadherin-expressing cells, we performed experiments using T24 cancer urothelial cells. 

It has been shown that cancer urothelial cells rarely attach to the highly differentiated urothelium but can attach to the traumatized urothelium where poorly differentiated urothelial cells of the basal or intermediate cell layers are exposed [13,30,31]. To maximize the adhesion of cancer urothelial cells to the urothelium, and thereby to maximize the number of attachment sites of interest, we used the *in vitro* model of the poorly differentiated urothelium. In such a urothelial model, only a few urothelial cells were uroplakin positive, while the majority of cells in the superficial layer were uroplakin negative. To more precisely evaluate the adhesion of T24 cells to the urothelium, we used an *in vitro* model of the normal partially differentiated urothelium in parallel. Our results show that T24 cancer urothelial cells only adhere to the poorly differentiated urothelial cells that are uroplakin negative, irrespective of their N-cadherin expression level. Since in the partially differentiated urothelium the majority of cells expressed uroplakins, the number of adhered cancer cell aggregates was accordingly much lower. How exactly uroplakins, or perhaps a layer of glycosaminoglycans, hinder the adhesion of cancer urothelial cells to the differentiated urothelial cells, remains unclear. 

Cadherins are among the key proteins that regulate cell–cell aggregation and cell motility. In epithelial cells, E-cadherins regulate base-apical cell polarity, however, in cancer cells, switching from E- to N-cadherin leads to more mesenchymal cell morphology and induction of their motility, collective migration, and invasiveness [16]. This is also why the cadherins are so extensively studied concerning cancer initiation and progression. Previous studies suggest that the adhesion of cancer urothelial cells to the urothelium could be E-cadherin-dependent [14,15]. The T24 and T24 Ncad^low^ cells do not express E-cadherin, but they can adhere to poorly differentiated urothelial cells. Moreover, our results show that lower expression of N-cadherin in the plasma membrane of T24 does not prevent their attachment to the poorly differentiated urothelial cells. Altogether this indicates that neither E- nor N-cadherin plays a decisive role in this adhesion. 

Both the T24 and T24 Ncad^low^ cells adhered to the urothelium in the form of cell aggregates. Due to the lower N-cadherin expression in the plasma membrane, the T24 Ncad^low^ interconnected into smaller and less compact aggregates than the T24 cells. At adhesion to the poorly differentiated urothelial cells, both the T24 and T24 Ncad^low^ cells formed lamellipodia and numerous filopodia. The attachment of cancer urothelial cells through the filopodia, as one of the first events of cancer urothelial cell adhesion to the urothelium, was also recently demonstrated by our research group on a model of orthotopic bladder tumor in mice [34]. We hypothesized that attachment of cancer urothelial T24 cells to the urothelium could be mediated by heterotypical E-N cadherin junctions. However, we have not shown co-localization of the E-N cadherin signals at the sites of the T24 cell attachment to the poorly differentiated urothelial cells. No adherens junctions were identified at the attachment sites between cancer urothelial cells and urothelial cells, nor on the ultrastructural level. Instead, the ultrastructural analysis of the attachment sites showed that both T24 and T24 Ncad^low^ adhered to the poorly differentiated urothelial cells by desmosomes. 

In cancer cells, the desmosomal proteins are usually downregulated, which indicates their increased invasive potential [35]. The T24 and T24 Ncad^low^ cells were shown to express desmoglein-2 and to interconnect with desmosomes in aggregates (Figure 2i,j). In the urothelium, the tight junctions prevent lateral diffusion of membrane proteins between apical and basolateral plasma membranes. In the poorly differentiated urothelium, the majority of the urothelial cells in the superficial layer do not interconnect with tight junctions, which may allow the desmosomal cadherins to diffuse to the apical surface of the cell where they are available for interaction with desmosomal cadherins of cancer urothelial cells. Nevertheless, in the partially differentiated urothelium, the superficial urothelial cells interconnect with tight junctions. To adhere to the partially differentiated urothelium by desmosomes, the T24 and T24 Ncad^low^ cells first disrupted tight junctions between superficial urothelial cells. We have shown that in monoculture the T24 and T24 Ncad^low^ cells secrete (pro) MMP-2 gelatinase, which was shown to cleave the tight junctional proteins [36,37,38]. In addition to MMPs, the disruption of the tight junctions could be also caused by hepatocyte growth factor [38,39] or interleukin-8 [40], both of which are upregulated in invasive bladder cancer [41,42]. To induce local disruption of the superficial urothelial cells’ tight junctions, the T24 or T24 Ncad^low^ cells should be in close contact with the superficial urothelial cells. This means that they are probably first anchored to the urothelium by other junctional proteins, e.g., the integrins, further proposing that desmosomes are secondary junctions in this interaction. However, the disruption of superficial urothelial cells’ tight junctions is required for the desmosomal cadherins to translocate from the basolateral to the apical position of the plasma membrane and interact with desmosomal cadherins of the T24 or T24 Ncad^low^ cells. 

Desmosomes are considered to be strong and stable cell–cell junctions. Nevertheless, emerging evidence indicates that desmosomes can also be fast and dynamic structures, and participate in cellular processes beyond that of cell adhesion [39]. Namely, Roberts et al. [40] showed that desmosomes can coordinate epithelial cell migration by rapidly assembling at the lateral edges between migrating epithelial cells, while if away from the leading edge they cluster and mature. In the epithelia, the homotypic interactions between classical cadherins mediate the initiation of the desmosome assembly [41,42]. Yet, the urothelial and T24 or T24 Ncad^low^ cells do not share any of the classical cadherins. How can these cells then form desmosomes? 

We hypothesize that the adhesion of T24 and T24 Ncad^low^ to the poorly differentiated urothelial cells is mediated through the desmosomal cadherins since they are the only type of cadherins that these cells have to interact with in homotypic fashion. Additionally, Shafraz et al. [42] used single-molecule atomic force measurements to show that desmoglein-2 can also interact with E-cadherin and form a Ca^2+^-independent heterodimer. They demonstrated that these two proteins can form trans (cell–cell) or cis (within a plasma membrane of a cell) heterodimers. Since T24 and T24 Ncad^low^ cells express desmoglein-2 and urothelial cells express E-cadherin, it might be that they also interconnect through these heterodimeric trans interactions. This study demonstrated the adherence of T24 and T24 Ncad^low^ cells to the uroplakin-negative urothelial cells in the 24 h time point. To determine the time frame of desmosome assembly more accurately and to identify the possible primary contacts between the T24 and the urothelial cells, the earlier time points of this adhesion mechanism should be evaluated in the future. 

## 4. Materials and Methods

### 4.1. Cell Cultures

As an *in vitro* model of poorly differentiated urothelium, we used human non-invasive cancer urothelial cells, RT4, isolated from transitional cell papilloma (ATCC^®^ HTB-2™), as previously described [43,44]. Briefly, RT4 cells were seeded onto 12-well culture inserts with porous membranes with 0.4-μm pores and 0.9-cm^2^ effective growth areas (BD Falcon, Franklin Lakes, NJ, USA) at a seeding density of 5 × 10^4^ cells/cm^2^ and cultured in medium A-DMEM-F12 for a week. The culture medium consisted of equal parts of A-DMEM (Gibco, Life Technologies, Thermo Fisher Scientific, Waltham, MA, USA) and F12 (HAM) (Sigma-Aldrich, Merck, Darmstadt, Germany), supplemented with 5% fetal bovine serum (FBS; Gibco, Life Technologies), 4 mM GlutaMAX (Gibco, Life technologies), and 1% penicillin-streptomycin solution (Gibco, Life technologies). 

As an *in vitro* model of the partially differentiated urothelium, we used normal porcine urothelial (NPU) cells. Primary and secondary NPU cells were established from two porcine urinary bladders, obtained independently from a local abattoir as described in [45,46]. The NPU cells were seeded onto 12-well culture inserts with porous membranes with 0.4-μm pores and 0.9-cm^2^ effective growth areas (BD Falcon), at a seeding density of 2 × 10^5^ cells/cm2 and cultured in UroM medium (i.e., UroM (−Ca^2+^ + S_FBS_)) adapted for normal urothelial cells. UroM (−Ca^2+^ + S_FBS_) consisted of equal parts of MCDB153 medium (Sigma-Aldrich) and advanced Dulbecco’s modified essential medium (Invitrogen, Life Technologies), supplemented with 2.5% FBS, 0.1 mM phosphoethanolamine (Sigma-Aldrich), 15 mg/mL adenine (Sigma-Aldrich), 0.5 mg/mL hydrocortisone (Sigma-Aldrich), 5 mg/mL insulin (Sigma-Aldrich), 2 mM GlutaMAX (Gibco, Life Technologies), and penicillin-streptomycin solution (100 U/mL of penicillin and 100 mg/mL streptomycin; Gibco, Life Technologies). Cell cultures were maintained at 37 °C and 5% CO_2_ in a 95% humidified atmosphere, until confluency, which was 5 to 7 days.

As an *in vitro* model of cancer urothelial cells, we used human invasive cancer urothelial cells, T24, isolated from transitional cell carcinoma (ATCC^®^ HTB-4™) and T24 Ncad^low^ cells, which were established *de novo* from the T24 cell line (described in detail in Section 4.2). The culturing of both cell lines is described in detail in Section 4.3). 

Platinum-GP cells (Cell Biolabs, RV-103) were cultured in Dulbecco’s modified essential medium (Gibco, Life Technologies) supplemented with 10% FBS.

### 4.2. Establishment of Cancer Urothelial Cell Line T24 with Low Expression of N-Cadherin

For the establishment of T24 Ncad^low^ cancer urothelial cells, we transduced T24 cancer urothelial cells with retroviruses encoding shRNA against N-cadherin. First, to produce retroviruses encoding shRNA against N-cadherin, the Platinum-GP packaging cells were transfected with pGFP-V-RS plasmid (OriGene, TG314034, Herford, Germany) and pCMV-VSV-G plasmid (Cell Biolabs, San Diego, CA, USA) by lipofection (Lipofectamine 2000, ThermoFisher Scientific). T24 cells were then transduced by retroviruses encoding shRNA against N-cadherin and selected using puromycin (10 µg/mL). After antibiotic selection, cells were single-cloned by limited-dilution protocol. The expression of N-cadherin in different cell clones was determined by Western blot and immunofluorescence of N-cadherin. For the performance of the aggregate adhesion experiments, only the cell clone which showed the most decreased expression of N-cadherin in the plasma membrane was used. 

### 4.3. Seeding of Cancer Urothelial Cells on the Poorly or Partially Differentiated Urothelium

For monitoring of cancer cell attachment to the urothelium, T24 and T24 Ncad^low^ cells were labeled with red fluorescent lipophilic dye (1:200; Vybrant DiI, ThermoFisher Scientific) for fluorescence microscopy, or with cobalt ferrite (CoFe_2_O_4_) nanoparticles coated with polyacrylic acid (PAA nanoparticles; 100 µg/mL) for transmission electron microscopy. Nanoparticles were synthesized and characterized as described by Bregar and colleagues [47]. Labelled T24 and T24 Ncad^low^ cells were seeded onto the confluent poorly or partially differentiated urothelium with a seeding density of 1 × 10^5^ cells/cm^2^. The cancer urothelial cells seeded on the poorly differentiated urothelia were cultured in the A-DMEM + F12 medium, whereas cancer urothelial cells seeded on the partially differentiated urothelium were cultured in the UroM (−Ca^2+^ + S_FBS_) medium. Twenty-four hours after their seeding, the non-attached cancer urothelial cells were removed by rinsing with a fresh culture medium. The urothelia with attached aggregates of cancer urothelial cells were fixed for fluorescence and electron microscopy. Using poorly differentiated urothelium, we conducted six independent experiments in quadruplets for T24 and two independent experiments in quadruplets for T24 Ncad^low^ cells. Whereas, using partially differentiated urothelium, we performed ten independent experiments in at least sextuplets for T24 cells and four independent experiments in at least sextuplets for T24 Ncad^low^ cells. 

### 4.4. Quantitative Analysis of the Cancer Urothelial Cell Aggregates Adhered to the Poorly or Partially Differentiated Urothelium

Immediately after rinsing of non-attached cancer urothelial cells, the poorly or partially differentiated urothelia with attached aggregates of T24 and T24 Ncad^low^ cells were examined with a fluorescence microscope and phase-contrast microscope Eclipse TE300 (Nikon, Tokyo, Japan), depending on whether cancer urothelial cells were labelled with fluorescent lipophilic dye or with PAA nanoparticles. In the case of poorly differentiated urothelia, we acquired images of the equally large area (1.15 mm^2^) of the poorly differentiated urothelia with adhered aggregates of cancer urothelial cells (T24 cells (N = 95), T24 Ncad^low^ (N = 45). The sites of imaging were selected randomly. The aggregates of cancer urothelial cells attached to the urothelium were counted using the Cell Counter plugin of the ImageJ software and their number was expressed per cm^2^, presuming their uniform distribution. In the case of the partially differentiated urothelium, we counted all the T24 and T24 Ncad^low^ aggregates that were attached to the urothelium of 0.9 cm^2^ area. 

The evaluation of the cancer cell aggregate size was analyzed using ImageJ software (8-bit type images, Adjust Threshold, Analyze, Analyze particle plugin). We analyzed 1917 T24 cell aggregates and 1175 cell T24 Ncad^low^ aggregates adhered to poorly differentiated urothelium and 13 T24 cell aggregates and 19 T24 Ncad^low^ cell aggregates adhered to partially differentiated urothelium. The size of cell aggregates was expressed in µm^2^ of the surface area. 

### 4.5. Immunofluorescence Analysis

All established *in vitro* models were fixed in 4% paraformaldehyde in PBS for immunolabelling of E- and N-cadherin, or in ice-cold absolute ethanol for immunolabelling of uroplakins and occludin. After fixation, the samples were washed in PBS and blocked in blocking buffer (0.1% gelatin, 0.1% saponin, 0.5% bovine serum albumin (BSA), and 50 mM NH_4_Cl in 0.02% NaN_3_) in the case of fixation in 4% paraformaldehyde, or in 1% BSA in PBS in the case of fixation in absolute ethanol, both at room temperature for 1 h. The samples were then incubated at 4 °C overnight with primary antibodies as follows: rabbit polyclonal antibodies against N-cadherin (1:100, ab12221, Abcam, Cambridge, United Kingdom), mouse monoclonal antibodies against E-cadherin (1:400, 610182, BD-Pharmingem, CA, United States), rabbit polyclonal antibodies against uroplakins (1:1000; a gift from Prof. T.T. Sun), and rabbit polyclonal antibodies against occludin (1:400; 71–1500, Invitrogen, Thermo Fisher Scientific), all diluted in 1% BSA in PBS. For negative controls, the primary antibodies were omitted and samples were incubated in 1% BSA in PBS at 4 °C overnight. After washing in PBS, samples were incubated with appropriate secondary antibodies: goat anti-mouse (Alexa Fluor 488 or Alexa Fluor 555; Invitrogen, Molecular Probes, Thermo Fisher Scientific) or goat anti-rabbit (Alexa Fluor 488; Invitrogen, Molecular Probes, Thermo Fisher Scientific), at room temperature for 1.5 h. The secondary antibodies were diluted 1:400 in 1% BSA in PBS. After washing in PBS, the samples were mounted in Vectashield mounting medium with 4′,6-diamidino-2-phenylindole (DAPI) (Vector Laboratories, Burlingame, CA, USA) for DNA labelling. The samples were analyzed with a fluorescence microscope AxioImager.Z1 equipped with ApoTome (Carl Zeiss MicroImaging GmbH, München, Germany).

### 4.6. Transmission and Scanning Electron Microscopy 

For ultrastructural analysis of the cancer urothelial cell attachment to the urothelium, we performed transmission and scanning electron microscopy. For transmission electron microscopy all *in vitro* models were fixed in 3% (*w*/*v*) paraformaldehyde and 3% (*v*/*v*) glutaraldehyde in a 0.1 M cacodylate buffer, pH 7.4 for 3 h at 4 °C. The fixation was followed by overnight rinsing in the 0.1 M cacodylate buffer at 4 °C and post-fixed in 2% (*w*/*v*) osmium tetroxide for 1 h at room temperature. The samples were then dehydrated in a graded series of ethanol and embedded in Epon (Serva Electrophoresis, Heidelberg, Germany). Ultrathin sections were contrasted with uranil acetate and lead citrate and examined with a transmission electron microscope (Philips CM100, Tokyo, Japan) equipped with AMT camera (Advanced Microscopy Techniques Corp., Woburn, MA, USA). 

For scanning electron microscopy, all *in vitro* models were fixed in 2% (*w*/*v*) paraformaldehyde and 2% (*v*/*v*) glutaraldehyde in a 0.2 M cacodylate buffer, pH 7.4 for 3 h at 4 °C. The samples were rinsed in 0.2 M cacodylate buffer overnight at 4 °C and post-fixed in 1% osmium tetroxide in the same buffer for 2 h at room temperature. After rinsing in 0.2 M cacodylate buffer and dehydration in a graded series of ethanol, the samples were completely dehydrated in acetone and hexamethyldisilazane (HMDS) (Sigma-Aldrich). The dehydrated samples were spattered with gold and examined with a scanning electron microscope (Tescan Vega 3, Brno, Czech Republic).

### 4.7. In Gel Gelatin Zymography

To detect the metalloproteinases secreted by T24 and T24 Ncad^low^ cells, we performed in gel gelatin zymography (first described by [48]). For this, cancer urothelial cells were seeded on tissue culture flasks (TPP, Trasadingen, Switzerland) at a density of 5 × 10^4^ cells/cm2. Cells were grown in the growth medium adapted for cancer urothelial cells, until sub-confluency. The cells were then washed with sterile PBS, and grown in the FBS-free growth medium, for an additional 24 h. Afterwards, the growth medium was collected, centrifuged (10 min, 200g, 4 °C), and the supernatants were frozen at −80 °C. The protein concentration in the samples was determined by the BCA method (ThermoFisher Scientific). The samples with the final concentration of 5 μg proteins/μL were separated by 10% SDS-polyacrylamide electrophoresis (SDS-PAGE) on gels containing 0.1% gelatin, at 4 °C. The recombinant gelatinases MMP-2 (ab81550, Abcam) with the final concentration of 0.5 ng/μL were loaded as protein standards. Afterwards, the gels were rinsed in distilled water and incubated in the renaturation protein buffer (2.5% Triton X-100 in distilled water), with gentle agitation, twice for 30 min. After the rinsing of gels with distilled water and incubation in the developing buffer (0.5 M Tris HCl (pH 7.8), 2M NaCl, 0.05 M CaCl_2_, 0.2% Triton X-100 in distilled water) for 22 h, at 37 °C, the gels were stained with Coomassie blue (Bio-Rad Laboratories, Hercules, CA, USA, 0.5% Coomassie blue, 5% methanol, 10% acetic acid in distilled water) for an hour and destained in destaining solution (5% ethanol, 10% acetic acid in distilled water). The gelatin zymography was performed three times independently. 

### 4.8. Cell Surface Biotinylation

To isolate the plasma membrane fraction of the T24 and T24 Ncad^low^ cells, we performed cell surface biotinylation (first described by [49]). For this, cancer urothelial cells T24 and T24 Ncad^low^ at 80% confluency were washed with ice-cold PBS and labelled with EZ-Link Sulfo-NHS-SS-Biotin (89881, Thermo Fisher Scientific) at 4 °C for 30 min, following the manufacturer’s instructions. In brief, quenching solution was added and cells washed with TBS, scrapped, and centrifugated (500× *g*, 3min). Cell pellets were lysed with lysis buffer and biotinylated proteins isolated in NeutrAvidin Gel. Proteins were eluted by SDS-PAGE sample buffer with 50mM DTT. Proteins were subsequently analyzed by Western blot as described below. The gray values of band densities (N of loadings = 5) were analyzed semi-quantitatively with Fiji program. The results are presented as an average of band densities of N-cadherin at the surface of T24 and T24 Ncad^low^ cells per band densities of plasma membrane marker ENaCγ in T24 and T24 Ncad^low^ cells (in arbitrary units).

### 4.9. Western Blot 

For Western blot, NPU cells, poorly differentiated urothelial cells RT4, and cancer urothelial cells T24 and T24 Ncad^low^ were washed and scraped into cold sterile PBS. The cell suspensions were centrifuged (10 min, 200× *g*, 4 °C). Pellets were lysed with RIPA buffer (150 mM NaCl, 50 nM Tris (pH 8), 1 mM EDTA, 1% Triton, and 0.1% SDS in 0.5% sodium deoxycholate in distilled water) supplemented with 100× Halt™ Protease Inhibitor Cocktail (Thermo Fisher Scientific), for 30 min, on ice. Samples were then centrifuged (10 min, 15.5000× *g*, 4 °C) and protein concentration determined by the BCA method (ThermoFisher Scientific). Samples were diluted with sample buffer supplemented with DTT (Sigma-Aldrich) (*v*/*v*, 1:4). 8 µg of proteins per lane were loaded and separated on the 4–20% Novex tris-glycine gels (Thermo Fisher Scientific) and transferred on nitrocellulose membranes (Amersham Biosciences, Amersham, U.K.). The membranes were blocked in 5% milk in PBS supplemented with 0.1% Tween-20 (T-PBS) (Sigma-Aldrich) for one hour, at room temperature, and then incubated at 4 °C overnight with monoclonal mouse antibodies against E-cadherin (610182, 1:1000, BD Transduction Laboratories, San Jose, CA, USA), polyclonal rabbit antibodies against N-cadherin (ab18203, 1:400, Abcam), polyclonal rabbit antibodies against desmoglein 2 (ab 76668, 1:100, Abcam), and polyclonal rabbit antibodies against Epithelium Sodium Channel γ (ENaCγ, ab65707, 1:1000, Abcam), all diluted in T-PBS. The primary antibodies were detected using anti-mouse or anti-rabbit polyclonal secondary antibodies conjugated with horse-radish-peroxidase (1:1000, Sigma-Aldrich). Chemiluminescence signals were visualized with LAS-4000 CCD camera (Fujifilm; Tokyo, Japan). 

The intensity of Western blot bands from biotinylated samples was analyzed with Fiji program (N = 5 for T24 and N = 5 for T24 Ncad^low^ cells). 

### 4.10. Hanging Drop Assay

To evaluate the tightness of intercellular junctions in T24 and T24 Ncad^low^ cell aggregates, we performed hanging drop assay (adapted from [50]). For this, cancer urothelial cells T24 and T24 Ncad^low^ were harvested with TripleSelect (Gibco, Life Technologies), counted using a haemocytometer, and adjusted to a concentration of 2 × 10^6^ cells/mL. 30 µl drops of cell suspension were deposited on the bottom of the lid of the 100 mm cell culture dish and the corresponding culture dish was filled with 10 mL sterile PBS. The PBS-filled culture dish was then covered with the lid, which resulted in hanging drops of cell suspension from the lid. Cells were incubated at 37 °C in a 95% humidified atmosphere of 5% CO2 in the air until cell aggregates have formed at the bottom of the drop (1–2 days). Cell–cell adhesion was assessed by counting the cells released from the cell aggregates after forceful pipetting (10 times with a 200 μL Eppendorf pipette tip cut widely 5–6 mm from the tip). For each of the cell lines, we conducted at least four independent experiments in quadruplicates.

### 4.11. Statistical Analysis

Presented data are expressed as mean ± standard error of the mean (SEM). Statistical analysis was performed using a non-parametric Mann–Whitney test, or a two-tailed Student’s t-test where applicable. *p*-values of < 0.05 were considered statistically significant.

## 5. Conclusions

Bladder cancer is often multifocal and has a high recurrence rate. Whether the multifocal or recurrent tumors result from the premalignant genetic modifications already present in the urothelium, the dissemination and reimplantation of the cancer urothelial cells, or a combination of both, remains elusive. The presented study supports the intraluminal dissemination of bladder cancer cells. We have shown that N-cadherin does not play a crucial role in the attachment of T24 cells to the urothelium. Namely, cancer urothelial cells T24 and T24 Ncad^low^ with lower expression of N-cadherin in the plasma membrane both adhere to the poorly differentiated urothelial cells by desmosomes. Understanding the cellular mechanisms involved in the adhesion and spreading of cancer urothelial cells along the urothelium is crucial for the successful treatment of urinary bladder cancer.

## 6. Patents

The *in vitro* model of partially differentiated urothelium with attached T24 cancer urothelial cells is patent protected in Slovenia (Patent No. SI 23673). 

## Data Availability

Not applicable.
